# Frailty in Older Adults with Severe Aortic Stenosis: The Role of Systemic Inflammation and Calcium Homeostasis

**DOI:** 10.3390/jcm14020334

**Published:** 2025-01-08

**Authors:** Pablo Solla-Suarez, Marta Encuentra-Sopena, Marcel Almendárez, Rut Álvarez-Velasco, Tatiana Martin-Vega, Pablo Avanzas, Eva López-Álvarez, Ana Coto-Montes, José Gutiérrez-Rodríguez

**Affiliations:** 1 Geriatrics Department, Geriatrics Clinical Management Area, Monte Naranco Hospital—Central University Hospital of Asturias, 33011 Oviedo, Spain; 2 Health Research Institute of Asturias, ISPA, 33011 Oviedo, Spain; 3 Cardiology Department, Cardiac Area, Central University Hospital of Asturias, 33011 Oviedo, Spain; 4 CIBER Cardiovascular Diseases (CIBERCV), 28029 Madrid, Spain; 5 Faculty of Medicine and Health Sciences, University of Oviedo, UOV, 33006 Oviedo, Spain; acoto@uniovi.es; 6 Institute of Neurosciences of the Principality of Asturias, INEUROPA, 33006 Oviedo, Spain

**Keywords:** frailty, aortic valve stenosis, inflammation, interleukin-6, calcium homeostasis

## Abstract

**Background:** Frailty and severe aortic stenosis (AoS) are critical conditions in older adults, both of which share pathophysiological mechanisms including chronic inflammation and calcium metabolism dysregulation, potentially influencing the development and progression of these conditions. This study aimed to analyze systemic inflammation and calcium homeostasis biomarkers and their associations with frailty in older adults with severe AoS. **Methods**: This prospective study included 191 patients aged ≥75 years with severe AoS who were candidates for aortic valve replacement and were evaluated at a Geriatrics Frailty Assessment and Intervention Clinic. Frailty was defined as a score ≤6 on the Short Physical Performance Battery (SPPB). Biomarkers analyzed included aortic valve calcium score, parathyroid hormone (PTH), calcidiol (vitamin D), calcium, phosphate, creatinine, interleukin-6 (IL-6), and the Systemic Immune-Inflammation Index. Multivariate logistic regression was performed to identify independent predictors of frailty. **Results**: Of the 191 patients studied, 53.9% were women, with a mean age of 84.1 ± 4.1 years. Frailty was identified in 28.3% of patients (mean SPPB score 7.6 ± 2.5). Statistically significant differences between frail and non-frail patients were observed for PTH (87.7 ± 61.1 pg/mL vs. 70 ± 44.4 pg/mL, *p* = 0.028) and IL-6 (10.4 ± 11.2 pg/mL vs. 7 ± 8.2 pg/mL, *p* = 0.049). Notably, in the multivariate model, IL-6 emerged as a significant independent predictor of frailty (OR 1.037; CI 1.001–1.074, *p* = 0.043). **Conclusions**: IL-6 was identified as a biomarker significantly associated with frailty in older adults with severe AoS. Evaluating IL-6 could enhance the precision of frailty assessments, complement functional measures, and support clinical decision-making in this population.

## 1. Introduction

The prevalence of aortic stenosis (AoS) increases with age and is primarily of a degenerative origin, especially in older adults [1]. The disease progresses through a subclinical phase characterized by cellular changes at the valve level and a proinflammatory state, eventually leading to aortic valve calcification, cardiac remodeling, and hemodynamic impairment in the symptomatic phase [2].

The indication of treatment for AoS with normal left ventricular function requires the existence of severe and symptomatic valve disease, since it is from this point when the risk of clinical worsening, functional deterioration, and death increases drastically [3]. The optimum time window for treatment has been studied in clinical trials analyzing the results of earlier intervention in patients with severe AoS but no symptoms [4]. Current research focuses on identifying therapeutic targets by exploring the mechanisms underlying the pathophysiology of the disease, since to date no medical treatment has been shown to be effective in reverting, arresting, or slowing calcification and valve stenosis [5].

At present, the only available disease-modifying treatments are surgical aortic valve replacement or transcatheter (percutaneous) aortic valve replacement. The latter technique is less aggressive and is therefore more often used in patients over 75 years of age, since these individuals generally have a high surgical risk and other important non-cardiological comorbidities. Frailty is characterized by a systemic decrease in the response to stressors associated with advanced age, and has been shown to play a key role in this context, as it is a predictor of mortality and disability after aortic valve replacement (AVR) therapy [6].

The usefulness of different instruments for detecting frailty in patients subjected to valve replacement has been widely established [7]. However, the existence of multiple strategies for assessing frailty results in a lack of uniformity in the recordings, with marked variations in prevalence depending on the series. Biomarkers are thus needed to allow a more precise and uniform characterization of frailty [8].

The identification of biomarkers to complement point-of-care assessments of frailty has become essential, particularly in scenarios where functional evaluations are critical for decision-making and where targeted rehabilitation interventions may improve patient outcomes. In this context, the opportunistic detection of osteosarcopenia through computed tomography (CT) imaging performed before transcatheter AVR has demonstrated its potential to provide valuable clinical insights [9]. Additionally, systemic biomarkers, such as elevated C-reactive protein, a well-established marker of inflammation, have been linked to muscle catabolism and functional decline in this population [10].

Despite these advances, the integration of circulating biomarkers into routine clinical decision-making remains underexplored, particularly regarding their predictive value for adverse health outcomes in older adults with AoS. This highlights a need for additional research to refine their application, establish reliable cut-off points, and clarify their role in guiding the management of this vulnerable population. Addressing these gaps through more high-quality studies is crucial to enhance the predictive accuracy of these biomarkers and ultimately improve clinical outcomes [11].

An important avenue for advancing this field involves the investigation of specific biological pathways potentially underlying both frailty and AoS. Chronic inflammation and alterations in calcium metabolism are pathophysiological factors associated with frailty and must be taken into account in identifying biological markers of the disorder [12]. In addition, the potential modulating role of these pathways—which are also implicated in the development of AoS—in the capacity of response to stress has not been studied in depth in older adults with this valve disease.

The primary objective of the present study was to analyze systemic inflammation and calcium homeostasis biomarkers and their potential associations with frailty in a cohort of older patients with severe AoS amenable to AVR.

## 2. Materials and Methods

### 2.1. Study Design and Participants

A prospective study was carried out in patients 75 years of age or older with severe AoS belonging to the FRESAS (FRailty Evaluation in Severe Aortic Stenosis) cohort. The patients were consecutively referred to the only cardiac valve Heart Team in the region (Spanish Autonomous Community) of Asturias, and were considered to be potential candidates for either surgical or transcatheter AVR.

All patients were recruited for the study and evaluated in a specialized clinic for the detection of frailty and prehabilitation in older patients with severe AoS amenable to AVR prior to the intervention.

The inclusion criteria were (i) a diagnosis of severe AoS; (ii) age 75 years or older; and (iii) the provision of written informed consent to participate in the study. The exclusion criterion was the presence of active infection at the time of patient evaluation, due to its potential impact upon the values of most of the main biomarkers analyzed in the study. Two patients failing to give consent and another presenting active infection were excluded from the study.

Patients were recruited over a two-year period, from June 2021 to May 2023.

### 2.2. Study Variables

i. Comprehensive Geriatric Assessment

All patients underwent a Comprehensive Geriatric Assessment (CGA), including variables of its main domains: clinical (cardiological parameters, other diseases, short-form Charlson comorbidity index, and polypharmacy), functional (frailty, Barthel index of dependency for basic activities of daily living [BADL], and Lawton index of dependency for instrumental activities of daily living [IADL]), nutritional (Mini-Nutritional Assessment—Short Form), cognitive (Mini-Mental State Examination [MMSE]), and mood state (Yesavage depression scale).

ii. Frailty

The functional assessment included a detailed evaluation of frailty. The Short Physical Performance Battery (SPPB) was used for this purpose, defining frailty as a score of ≤6. This threshold is well established as a reliable indicator of frailty, strongly associated with an increased risk of sarcopenia and mortality in older adults [13,14]. Furthermore, an SPPB score of ≤6 has been consistently applied in prior studies within our cohort, ensuring consistency with previous research on frailty in older patients with AoS [8].

iii. Systemic inflammation

The serum interleukin-6 (IL-6) levels were quantified by an electrochemiluminescence immunoassay (ECLIA) using a Cobas e801 system (Roche Diagnostics). In addition, the Systemic Immune-Inflammation Index (SII, in cells × 10^3^/mL) was calculated based on the formula [absolute platelet count × (absolute neutrophil counts/absolute lymphocyte count)].

iv. Calcium homeostasis

The aortic valve calcium score (in Agatston units [AU]) was quantified using CT. The main variables analyzed at the systemic level were calcidiol vitamin D (25OH-D3) and intact parathyroid hormone (PTH). Both parameters were likewise quantified via ECLIA using a Cobas e801 system (Roche Diagnostics). The following cut-off points were considered: 25OH-D3 < 20 ng/mL (deficiency) and <10 ng/mL (severe deficiency); PTH > 65 pg/mL (hyperparathyroidism).

Measurements of calcium (in mmol/L), phosphate (in mmol/L), and creatinine (in mg/dL) were also made.

The blood samples were collected using a predefined protocol in the CGA clinic before the physical performance tests were conducted.

### 2.3. Ethical Particulars

This study was conducted in observation of the international guidelines on clinical research (Declaration of Helsinki of the World Medical Association), and was approved by the local Research Ethics Committee (CEImPA, code 281-19).

### 2.4. Statistical Analysis and Interpretation of Results

Data were expressed as the mean ± standard deviation (SD) in the case of continuous quantitative variables, and as percentages and/or frequencies in the case of qualitative variables. The chi-square test and/or Fisher’s exact test were used to compare qualitative variables between groups, while quantitative variables were compared using the Student t-test or analysis of variance (ANOVA). The Pearson correlation coefficient (r) was used to evaluate associations between quantitative variables. In the case of variables lacking defined standardized reference values in which a comparative qualitative analysis was considered to be indicated, tertiles were used (“low”, “intermediate”, and “high” groups). Differences in the primary variable (frailty) between groups were explored by multivariate logistic regression analysis, using the backward-stepwise method. Statistical significance was considered for *p* < 0.005. Data analysis was performed using the SPSS version 25 statistical package.

## 3. Results

### 3.1. Baseline Characteristics

A total of 191 patients were included in this study: 53.9% women, with a mean age of 84.1 ± 4.1 years, and a mean EuroSCORE-II of 2.9 ± 1.8 points. With regard to AoS, the mean aortic valve area was 0.7 ± 0.2 cm^2^, with a mean pressure gradient of 45.3 ± 12.3 mmHg and a maximum pressure gradient of 75.2 ± 16.9 mmHg. The left ventricular ejection fraction was preserved in 89.5% of the patients, and 20.4% presented a New York Heart Association (NYHA) functional class of ≥3.

The most frequent previous disease conditions were arterial hypertension (74.9%), dyslipidemia (62.8%), atrial fibrillation (36.1%), anemia (33.5%), and heart failure (31.4%). Sixty-five patients (34%) had been previously diagnosed with vitamin D deficiency. The patients were prescribed an average of 8.2 ± 3.4 drugs (79.6% presented polypharmacy) and had a short-form Charlson index of 1.7 ± 1.4 points (comorbidity in 47.1%, ≥2 points).

In turn, 4.7% of the patients presented malnutrition as determined by the Mini-Nutritional Assessment—Short Form (score ≤ 7). On the other hand, 15.7% of the patients presented Mini-Mental State Examination scores consistent with cognitive impairment (≤24 points), and 29.3% had a depressive mood state according to the Yesavage depression scale (score ≥ 5).

### 3.2. Functional Status and Frailty

With regard to the functional assessment, 54 patients presented with frailty (28.3%), with a mean SPPB score of 7.6 ± 2.5. The mean Lawton index was 5.2 ± 2.1 (41.9% being dependent for IADL), with a mean Barthel index of 91.9 ± 11 (38.7% being dependent to some extent for BADL). The stratification of the scores corresponding to the Functional Continuum Scale was 1: 16.8% (robust and independent for IADL), 2: 6.8% (robust but dependent for IADL), 3: 18.8% (pre-frail and independent for IADL), 4: 20.9% (pre-frail but dependent for IADL), 5: 13.1% (frail and independent for BADL), 6: 22% (slightly dependent for BADL), 7: 1% (moderately dependent for BADL), 8: 0.5% (severely dependent for BADL). No significant differences were observed on analyzing age between groups (age in frail patients 84.5 ± 4.2 years versus 84 ± 4.1 years in non-frail patients; *p* = 0.496).

### 3.3. Frailty Biomarkers—Univariate Analysis

The mean values of the studied biomarkers and the differences according to patient frailty are shown in Table 1. Statistically significant differences were found in the univariate analysis for PTH (70 ± 44.4 pg/mL in non-frail versus 87.7 ± 61.1 pg/mL in frail patients; *p* = 0.028) and IL-6 (7 ± 8.2 pg/mL in non-frail versus 10.4 ± 11.2 pg/mL in frail patients; *p* = 0.049). Likewise, hyperparathyroidism (>65 pg/mL) was found to be more prevalent in frail patients than in non-frail individuals (59.3% versus 40.1%; *p* = 0.017), in the same way as high IL-6 (>6 pg/mL, identifying p66 as upper tertile: 7 pg/mL) (55.6% versus 32.8%, *p* = 0.004) in frail and non-frail patients, respectively.

On examining possible differences in functional performance and normal parathyroid function or hyperparathyroidism (Table 2), differences were only observed in the mean SPPB score (8 ± 2.5 pg/mL in patients with normal PTH versus 7.2 ± 2.4 pg/mL in patients with hyperparathyroidism; *p* = 0.019). No statistically significant differences were observed in relation to the rest of the studied functional variables or in terms of age. A sub-analysis was performed, excluding patients with an estimated glomerular filtration rate (eGFR) <45 mL/min/1.73 m^2^ (*n* = 144, Supplementary Table S1), with statistical significance being reduced to a trend as a result (SPPB in patients with normal PTH 8.1 ± 2.6 versus 7.3 ± 2.5 in patients with hyperparathyroidism; *p* = 0.056). Another sub-analysis was performed, excluding patients with 25-OHD3 <20 ng/mL (*n* = 103, Supplementary Table S2), with statistically significant differences being found not only in SPPB (8.4 ± 2.3 in patients with normal PTH versus 7.4 ± 2.3 in patients with hyperparathyroidism; *p* = 0.031) but also in the Barthel index for BADL (94.7 ± 9.5 in patients with normal PTH versus 89.9 ± 10.8 in patients with hyperparathyroidism; *p* = 0.019). Significant differences in calcidiol levels were observed between seasonal periods: spring–summer (April–September), 23.7 ± 14.6 ng/mL, and autumn–winter (October–March), 19.7 ± 11 ng/mL (*p* = 0.031). No statistically significant seasonal differences were identified for PTH or any of the other biomarkers studied (Supplementary Table S3).

With regard to the analysis of differences according to frailty status and the quantification of high IL-6 levels (Table 3), differences were observed for all the studied parameters. Accordingly, patients with levels >6 pg/mL had lower SPPB, Lawton and Barthel scores and higher scores on the Functional Continuum Scale (*p* ≤ 0.001), reflecting a poorer functional condition. Significant differences in age between groups were likewise not found in this analysis.

The analysis of correlation among all the functional assessment parameters, including frailty (Table 4), showed that the greatest significant correlation corresponded to frailty versus IL-6 concentration, with r = −0.334 (*p* = 0.001). Only IL-6 concentration showed significant correlations with all the analyzed variables—higher IL-6 levels being associated with greater frailty and lesser functional capacity in terms of IADL and BADL (*p* < 0.005).

### 3.4. Frailty Biomarkers—Multivariate Analysis

A multivariate logistic regression analysis was conducted, including all the studied biomarkers, in order to analyze the variable frailty: aortic valve calcium score, PTH, calcidiol (vitamin D), calcium, phosphate, creatinine, IL-6. and SII. Only IL-6 was retained in the final model, being identified as a significant predictive biomarker of frailty, with an odds ratio (OR) of 1.037 (confidence interval [1.001–1.074] (*p* = 0.043)).

Consequently, a sub-analysis was performed to examine the potential relationship between IL-6 and the other biomarkers (Table 5). Elevated IL-6 levels were associated with significantly higher PTH levels (86.4 ± 68.7 pg/mL in elevated IL-6 compared to 67.6 ± 31.4 pg/mL in normal IL-6; *p* = 0.029). Similarly, creatinine levels were significantly higher in the elevated IL-6 group (1.2 ± 0.4 mg/dl compared to 1.0 ± 0.3 mg/dl in the normal IL-6 group; *p* = 0.004). Additionally, the Systemic Immune-Inflammation Index (SII) was significantly higher in patients with elevated IL-6 levels (956.8 ± 836.3 C·10^3^/mL compared to 660.1 ± 470.4 C·10^3^/mL in the normal IL-6 group; *p* = 0.006).

## 4. Discussion

In the present study of calcium homeostasis and systemic inflammation biomarkers, frailty in this cohort of older patients with symptomatic severe AoS was seen to be particularly associated with the serum concentration of IL-6. A less pronounced association was also found between frailty and the PTH levels. The mechanisms underlying chronic inflammation and calcium homeostasis have been shown to participate in the development of frailty. These mechanisms are also involved in the pathophysiology of AoS, highlighting their interconnection. This is the first study to globally assess these aspects, specifically in older patients with symptomatic severe AoS, from a CGA-focused perspective, as stated below.

Beyond its “classical” function in maintaining muscle and bone homeostasis, an increasing number of studies are exploring the systemic implications of the intact PTH (iPTH)–vitamin D hormone axis [15]. Its role as an effector of the musculoskeletal system could explain the possible associations previously found by other authors between low vitamin D levels and high PTH concentrations and an increased risk of developing frailty [16,17], representing one of the main systemic implications of the mentioned axis. Although evidence linking elevated PTH levels to frailty in older adults remains limited, it is hypothesized that PTH alters the function of both bone and muscle through mechanisms such as promoting protein catabolism and disrupting calcium metabolism. Nevertheless, although vitamin D has already been identified as a potential endocrine factor in the development of frailty, the benefits of supplementary therapies aimed at reversing frailty remain unclear [18]. In fact, most experts in this field agree that more evidence is needed, and controversial aspects remain regarding even the adequate sufficiency levels of vitamin D [19]. This may be because the maintenance of calcium homeostasis is highly complex, involving different modulators and clinical scenarios. Studies in this field require the joint evaluation of PTH, in view of its decisive role in regulating bone resorption and reabsorption, and in maintaining adequate serum calcium levels. In the present study, establishing cut-off points of <20 ng/mL for vitamin D deficiency and <10 ng/mL for severe deficiency, the observed prevalences were 53.9% and 16.9%, respectively. On the other hand, hyperparathyroidism was detected in 45.5% of the patients, and hyperparathyroidism associated with vitamin D deficiency was recorded in 28.3% of the subjects. Although no association was found between vitamin D levels and frailty considered in isolation, frailty was seen to be more prevalent in patients with hyperparathyroidism. This association tended to persist on separately analyzing individuals with vitamin D deficiency associated with hyperparathyroidism, and also on excluding those with impaired renal function. Our analysis revealed statistically significant seasonal variations in calcidiol levels, but no significant seasonal differences were found for PTH or any biomarkers studied. This suggests that the seasonal fluctuations in calcidiol levels may not substantially affect other markers of systemic inflammation or calcium homeostasis.

Comparatively, a study involving younger individuals with cardiac disease (mean age close to 70 years) found high PTH and low vitamin D levels to be associated with the presence of AoS. Accordingly, individuals with this valve disease exhibited higher PTH levels (51 pg/mL) and lower vitamin D concentrations (32 nmol/L or 12.8 ng/mL) [20]. Another study in older patients (mean age close to 80 years) and severe AoS reported a higher prevalence of vitamin D deficiency than in our cohort (38.7%) [21]. In a series of older individuals with AoS (mean age likewise close to 80 years), it was seen that up to two-thirds had low 25-OH vitamin D levels (<25 nmol/L or 10 ng/mL), but associated secondary hyperparathyroidism was present in only 20% [22]. This and other studies evidence the growing knowledge of the mechanisms involved in the development and progression of AoS. In this respect, it is accepted that both the activity of the osteoblasts in the valve tissue and of the osteoclasts in bone appear to be important in the development of the disorder, with potential shared mechanisms in the regulation of bone turnover and valve calcification. It is therefore of great interest to conduct further in-depth studies of the mechanisms participating in calcium metabolism and which modulate AoS, and to search for therapies that act upon calcium metabolism, especially considering the poor prognosis of this valve disease in this particular population [23]. Despite the extensive research on the role of key determinants of calcium metabolism in the pathophysiology of AoS, no studies to date have directly examined the relationship between PTH and frailty in older adults with aortic valve disease. Thus, further research is needed to better understand this relationship and its potential implications for the management and treatment of frailty within this clinical context.

Moreover, chronic inflammation has also been identified as a pathophysiological mechanism found in both frailty and AoS [24]. The scientific evidence in this respect remains scarce, however, particularly considering the strong impact of the former upon the latter, since frail individuals more often experience adverse outcomes with invasive treatments such as those commonly used for valve disease in its more severe stages. Both IL-6 [25] and SII [26] are chronic systemic inflammation biomarkers that have been identified as potential prognostic indicators in cardiovascular patients [27,28]. There is compelling evidence suggesting a potentially causal role of IL-6 signaling in frailty syndrome, with its association to several key features, including chronic low-grade inflammation, muscle dysfunction, and age-related disorders [29]. Furthermore, IL-6-mediated inflammation has been implicated in aortic calcification and in the development of valve disease [30,31]. In evaluating the SII, no significant differences were observed in the prevalence of frailty or in the functional performance scores among the individuals of our cohort. However, of the different studied markers, IL-6 showed the strongest association with frailty. Thus, the frail patients presented a mean IL-6 level of 10.4 pg/mL, which was significantly higher than in the non-frail patients, and a significant inverse correlation was moreover observed with the SPPB score. This association was confirmed by regression analysis including all the studied biomarkers. The subgroup of patients with elevated IL-6 levels also exhibited higher levels of PTH and creatinine. IL-6, produced in response to PTH, has been previously identified as a potential inducer of bone resorption [32]. Similarly, chronic kidney disease has been associated with elevated IL-6 levels [33]. Additionally, the observation of elevated SII levels in these patients aligns with the role of the SII as a marker of systemic inflammation. However, despite this association, our study did not find a correlation between the SII and frailty. This lack of correlation may reflect both the complexity of frailty and the role of systemic processes mediated by IL-6 [34]. Another research group analyzed IL-6 in patients with severe AoS previously subjected to transcatheter AVR, and reported no significant differences according to frailty—though the latter was assessed on a subjective basis, identifying those patients with limited mobility [35]. In our study, high IL-6 levels were consistently associated with objective functional assessment parameters, in addition to the SPPB score and the Lawton and Barthel indexes, with the recording of scores suggestive of functional deterioration in all cases.

Our research group has already studied different biological processes that exert a significant influence upon the increase in frailty among older adults, such as systemic oxidative stress and inflammation [36,37]. At clinical level, and specifically in older and oldest-old individuals with symptomatic severe AoS amenable to AVR, we have shown the importance of performing exhaustive functional evaluations based on CGA to optimize the treatment strategy through the detection of problems that can be intervened upon and potentially reverted, such as frailty [38]. We have also shown that the progressive loss of response to stressors in the course of the functional continuum (not only considering frailty as an isolated interval representative of the entire process) is an independent predictor of mortality at one year in these patients—a fact that may be useful in guiding treatment [39]. Thus, in recent years we have proposed the identification of objective biomarkers capable of improving precision in estimating the stress response capacity of older patients, particularly in situations where such estimation proves more complicated. The results of this study evidence that IL-6 could be regarded as one such biomarker, as it was seen to be associated with frailty in our cohort of older adults with severe AoS. In addition to its potential utility in identifying patients at higher risk for adverse outcomes, IL-6 could also serve as a valuable tool for monitoring the effectiveness of interventions aimed at reversing frailty.

The most important limitation of our study is its single-center design, which limits data extrapolation. To enhance generalizability, future studies should include multi-center data across different settings. Additionally, no sub-analyses were made taking into account supplementation with vitamin D and calcium, nutritional supplements, or other modulators of osteomuscular metabolism. Furthermore, the inherent characteristics of the cohort imply a low representation of individuals in the last stages of the functional continuum (with established moderate and severe dependency). All this implies that the association of IL-6, as a biomarker, with the loss of homeostasis that occurs over the entire continuum cannot be fully confirmed, and that further studies are therefore needed in this field.

The results of our study generate new research challenges in this setting. It seems necessary to further explore the role of IL-6, both as a potential predictor of health outcomes after AVR, and in monitoring the interventions proposed by the CGA to revert frailty, to optimize the outcomes. It also appears advisable to develop research lines allowing us to better understand how mechanisms associated with aging influence the course of valve disease.

## 5. Conclusions

Frailty is commonly observed in patients with severe AoS. It is potentially reversible and associated with increased vulnerability to external stressors such as AVR, resulting in a higher risk of complications following such treatment. Identifying frailty biomarkers is crucial to complement functional assessment of the patient. In the present study, IL-6 was found to be associated with frailty, and could be regarded as a biomarker of frailty in patients with severe AoS, offering the possibility of improved estimation of stress response in this clinical setting.

## Figures and Tables

**Table 1:** Biomarkers analyzed and differences according to frailty.

		Total	Frailty		
			No	Yes	
Variable	units	191 (100)	137 (71.7)	54 (28.3)	*p*-value
Calcium score	(AU)	3189.4 ± 1640.4	3242.1 ± 1693	3055.7 ± 1502.7	0.481
high	>3654 ^#^	63 (33) ^#^	48 (35)	15 (27.8)	0.216
PTH	(pg/mL)	75 ± 50.2	70 ± 44.4	87.7 ± 61.1	0.028 *
high	>65	87 (45.5)	55 (40.1)	32 (59.3)	0.017 *
Calcidiol	(ng/mL)	21.4 ± 12.7	21.4 ± 11.8	21.4 ± 14.9	0.995
Deficiency	<20	103 (53.9)	77 (56.2)	26 (48.1)	0.314
Severe deficiency	<10	24 (12.6)	14 (10.2)	10 (18.5)	0.119
Calcium	(mmol/L)	2.4 ± 0.1	2.4 ± 0.1	2.4 ± 0.1	0.461
Phosphate	(mmol/L)	1.1 ± 0.2	1.1 ± 0.2	1.1 ± 0.2	0.553
Creatinine	(mg/dL)	1.1 ± 0.3	1.1 ± 0.3	1.1 ± 0.3	0.772
IL-6	(pg/mL)	8 ± 9.3	7 ± 8.2	10.4 ± 11.2	0.049 *
high	>6 ^#^	75 (39.3) ^#^	45 (32.8)	30 (55.6)	0.004 *
SII	C·10^3^/mL	776.6 ± 653.8	747.9 ± 631.9	852.1 ± 706.9	0.318
high	>789 ^#^	64 (33.5) ^#^	44 (32.1)	20 (37)	0.517

Abbreviations: AU, Agatston units; PTH, intact parathyroid hormone; IL-6, interleukin-6; SII, Systemic Immune-Inflammation Index; C, cells. Results: mean and standard deviation (mean ± SD) or cases and percentage (*n* [%]). Considerations: (^#^) indicates variables analyzed using the upper tertile. (*) statistical significance (*p* < 0.05).

**Table 2:** Differences in functional capacity and age according to intact parathyroid hormone levels.

	PTH Levels (pg/mL)		
	Normal (≤65)	High (>65)	
Variable	104 (54.5)	87 (45.5)	*p*-value
Age	84.1 ± 4	84.1 ± 4.3	0.963
SPPB	8 ± 2.5	7.2 ± 2.4	0.019 *
Lawton	5.3 ± 2.2	5.1 ± 1.9	0.492
Barthel	93.3 ± 10.2	90.2 ± 11.7	0.052
Functional Continuum Scale	3.6 ± 1.8	4.1 ± 1.8	0.061

Abbreviations: PTH, intact parathyroid hormone; SPPB, Short Physical Performance Battery. Results: mean and standard deviation (mean ± SD). Considerations: (*) statistical significance (*p* < 0.05).

**Table 3:** Differences in functional capacity and age according to interleukin-6 (IL-6) levels.

	IL-6 Levels (pg/mL)		
	Normal (≤6)	High (>6)	
Variable	116 (60.7)	75 (39.3)	*p*-value
Age	84.4 ± 4.3	83.7 ± 3.7	0.287
SPPB	8.2 ± 2	6.8 ± 2.8	<0.001 *
Lawton	5.5 ± 1.9	4.6 ± 2.1	0.001 *
Barthel	94.1 ± 8.3	88.4 ± 13.5	<0.001 *
Functional Continuum Scale	3.4 ± 1.7	4.4 ± 1.7	<0.001 *

Abbreviations: IL-6, interleukin-6; SPPB, Short Physical Performance Battery. Results: mean and standard deviation (mean ± SD). Considerations: (*) statistical significance (*p* < 0.05).

**Table 4:** Correlation between the studied biomarkers and the functional and frailty assessment scales.

Variable	SPPB	Lawton	Barthel	FCS	
Calcium score	0.14	−0.291	0.114	−0.091	Pearson-r
	0.054	0.001 *	0.116	0.211	*p*-value
PTH	−0.155	−0.019	−0.111	0.12	Pearson-r
	0.032 *	0.796	0.127	0.099	*p*-value
Calcidiol	−0.035	0.121	0.001	0.052	Pearson-r
	0.628	0.096	0.989	0.473	*p*-value
Calcium	0.011	0.236	0.125	0.011	Pearson-r
	0.882	0.001 *	0.085	0.875	*p*-value
Phosphate	−0.062	0.176	−0.047	−0.015	Pearson-r
	0.398	0.015 *	0.522	0.837	*p*-value
Creatinine	−0.03	−0.094	0.051	0.031	Pearson-r
	0.684	0.196	0.481	0.666	*p*-value
IL-6	−0.344	−0.147	−0.189	0.206	Pearson-r
	0.001 *	0.043 *	0.009 *	0.004 *	*p*-value
SII	−0.154	−0.098	−0.111	0.111	Pearson-r
	0.034 *	0.176	0.126	0.127	*p*-value

Abbreviations: SPPB, Short Physical Performance Battery; FCS, Functional Continuum Scale; PTH, intact parathyroid hormone; IL-6, interleukin-6; SII, Systemic Immune-Inflammation Index. Considerations: (*) statistical significance (*p* < 0.05).

**Table 5:** Biomarker differences in relation to elevated interleukin-6 (IL-6) levels.

	IL-6 Levels (pg/mL)		
	Normal (≤6)	High (>6)	
Variable	116 (60.7)	75 (39.3)	*p*-value
Calcium Score (AU)	3044.3 ± 1544.1	3413.8 ± 1766.5	0.129
PTH (pg/mL)	67.6 ± 31.4	86.4 ± 68.7	0.029 *
Calcidiol (ng/mL)	21.0 ± 11.4	22.0 ± 14.6	0.597
Calcium (mmol/L)	2.4 ± 0.1	2.4 ± 0.1	0.097
Phosphate (mmol/L)	1.1 ± 0.2	1.1 ± 0.2	0.150
Creatinine (mg/dL)	1.0 ± 0.3	1.2 ± 0.4	0.004 *
SII (C·10^3^/mL)	660.1 ± 470.4	956.8 ± 836.3	0.006 *

Abbreviations: IL-6, interleukin-6; AU, Agatston units, PTH, intact parathyroid hormone; SII, Systemic Immune-Inflammation Index. C, cells. Results: mean and standard deviation (mean ± SD) or cases and percentage (*n* [%]). Considerations: (*) statistical significance (*p* < 0.05).

## Data Availability

The datasets analyzed during the current study are not publicly accessible. However, the data that support the findings of this study are available from the corresponding author upon reasonable request.

## Supplementary Materials

The following supporting information can be downloaded at: https://www.mdpi.com/article/10.3390/jcm14020334/s1, Table S1. Differences in functional capacity and age according to intact parathyroid hormone levels in patients with an estimated glomerular filtration rate (eGFR) ≥45 mL/min/1.73 m^2^ (*n* = 144); Table S2. Differences in functional capacity and age according to intact parathyroid hormone levels in patients with calcidiol 25OH-D3 <20 ng/mL (*n* = 103); Table S3. Analyzed biomarkers: differences according to seasonal periods.
