# Supplementary material for: Frailty in Older Adults with Severe Aortic Stenosis: The Role of Systemic Inflammation and Calcium Homeostasis
Source: J Clin Med. 2025 Jan 8;14(2):334. doi: 10.3390/jcm14020334 (PMC11766238; doi:10.3390/jcm14020334)
Supplement: Supplementary file 1 [file jcm-14-00334-s001.zip › jcm-3384078-supplementary.pdf]

Supplementary Table S1. Differences in functional capacity and age according to intact parathyroid hormone levels in patients with an estimated glomerular filtration rate (eGFR) ≥45 ml/min/1.73 m^2 (n = 144).

	**PTH levels** (pg/ml)		
	**Normal (≤65)**	**High (>65)**	
**Variable**	92 (63.9)	52 (36.1)	p-value
**Age**	84.1 ± 3.6	83.9 ± 4.3	0.798
**SPPB**	8.1 ± 2.6	7.3 ± 2.5	0.056
**Lawton**	5.2 ± 2.2	5 ± 1.9	0.564
**Barthel**	93 ± 10.6	91.2 ± 9.2	0.284
Functional Continuum Scale	3.5 ± 1.8	4 ± 1.7	0.166

Abbreviations: PTH, intact parathyroid hormone; SPPB, Short Physical Performance Battery. Results: mean and standard deviation (mean ± SD).

Supplementary Table S2. Differences in functional capacity and age according to intact parathyroid hormone levels in patients with calcidiol 25OH-D3 <20 ng/ml (n = 103).

	**PTH levels** (pg/ml)		
	**Normal (≤65)**	**High (>65)**	
**Variable**	49 (47.6)	54 (54.2)	p-value
**Age**	83.7 ± 3.7	8379 ± 4.7	0.909
**SPPB**	8.4 ± 2.3	7.4 ± 2.3	0.031
**Lawton**	5.3 ± 2.2	5 ± 1.9	0.372
**Barthel**	94.7 ± 9.5	89.9 ± 10.8	0.019
Functional Continuum Scale	3.2 ± 1.7	4 ± 1.9	0.027

Abbreviations: PTH, intact parathyroid hormone; SPPB, Short Physical Performance Battery. Results: mean and standard deviation (mean ± SD).

Supplementary Table S3. Analyzed biomarkers: differences according to seasonal periods.

	**Seasonal periods**		
	**April-September**	**October-March**	
**Variable**	110 (57.6)	81 (42.4)	p-value
**Calcium Score** (AU)	3242.9 ± 1627.6	3116.8 ± 1665.1	0.601
**PTH** (pg/ml)	71.2 ± 38.4	80.2 ± 62.7	0.256
**Calcidiol** (ng/ml)	23.7 ± 14.6	19.7 ± 11.0	0.031 *
**Calcium** (mmol/l)	2.4 ± 0.1	2.4 ± 0.1	0.169
**Phosphate** (mmol/l)	1.1 ± 0.2	1.1 ± 0.2	0.752
**Creatinine** (mg/dl)	1.1 ± 0.3	1.1 ± 0.4	0.671
**IL-6** (pg/ml)	8.6 ± 10.7	7.5 ± 8.0	0.440
**SII** ($C \cdot 10^3$/ml)	791.6 ± 608.7	765.7 ± 687.7	0.787

Abbreviations: AU, Agatston units, PTH, intact parathyroid hormone; IL-6, interleukin-6; SII, Systemic Immune-Inflammation Index. C, cells. Results: mean and standard deviation (mean ± SD) or cases and percentage (n [%]). Considerations: (*) statistical significance (p<0.05).
